# Leptin receptor gene polymorphisms and sex modify the association between acetaminophen use and asthma among young adults: results from two observational studies

**DOI:** 10.1186/s12931-018-0892-y

**Published:** 2018-09-19

**Authors:** Ali H. Ziyab, Nandini Mukherjee, Ramesh J. Kurukulaaratchy, Hongmei Zhang, Susan Ewart, Hasan Arshad, Wilfried Karmaus

**Affiliations:** 1 Department of Community Medicine and Behavioral Sciences, Faculty of Medicine, Kuwait University, Kuwait City, Kuwait; 2 Division of Epidemiology, Biostatistics, and Environmental Health, School of Public Health, University of Memphis, Memphis, TN USA; 3 Clinical and Experimental Sciences, Faculty of Medicine, University of Southampton, Southampton, UK; 4 David Hide Asthma and Allergy Research Centre, Isle of Wight, UK; 5 College of Veterinary Medicine, Michigan State University, East Lansing, MI USA

**Keywords:** Asthma, Sex, Acetaminophen, Leptin, Epidemiology

## Abstract

**Background:**

Epidemiologic studies have demonstrated associations between acetaminophen use and asthma. This investigation sought to determine whether sex modifies the acetaminophen-asthma association and whether leptin (*LEP*) and leptin receptor (*LEPR*) gene polymorphisms modulate the sex-specific associations.

**Methods:**

Data from the Isle of Wight birth cohort (IOW; *n* = 1456, aged 18 years) and Kuwait University Allergy (KUA; *n* = 1154, aged 18–26 years) studies were analyzed. Acetaminophen use and current asthma were self-reported. Genotype information for eighteen polymorphisms in *LEP* and *LEPR* genes were available in the IOW study. Associations between acetaminophen use and asthma were stratified by sex and genotype. Poisson regression models with robust variance estimation were evaluated to estimate adjusted prevalence ratios (aPR) and 95% confidence intervals (CI).

**Results:**

Acetaminophen use was dose-dependently associated with an increased prevalence of current asthma in the IOW and KUA studies. In both studies, sex-stratified analysis showed that acetaminophen use was associated with asthma among males, but not in females (*P*_interaction_ <  0.05). Moreover, a sex- and genotype-stratified analysis of the IOW data indicated that acetaminophen was associated with asthma to a similar extent among males and females carrying two common alleles of *LEPR* polymorphisms. In contrast, among those carrying at least one copy of the minor allele of *LEPR* polymorphisms, the magnitude of association between acetaminophen use and asthma was pronounced among males (aPR = 6.83, 95% CI: 2.87–16.24), but not among females (aPR = 1.22, 95% CI: 0.61–2.45).

**Conclusions:**

The identified sex-related effect modification of the acetaminophen-asthma association varied across *LEPR* genotypes, indicating that the sex-specific association was confined to individuals with certain genetic susceptibility. If the acetaminophen-asthma association is causal, then our findings will aid susceptibility-based stratification of at-risk individuals and augment preventive public health efforts.

**Electronic supplementary material:**

The online version of this article (10.1186/s12931-018-0892-y) contains supplementary material, which is available to authorized users.

## Background

Asthma is the most common inflammatory chronic disorder of the lungs, affecting both children and adults [1, 2]. Consistent epidemiologic evidence suggests that exposure to acetaminophen (paracetamol), a commonly used over-the-counter antipyretic/analgesic medication, in the intrauterine environment, infancy, childhood, and/or adult life is associated with asthma development [3–9]. Moreover, acetaminophen use during adolescence has been linked with persistence of asthma [10] and undiagnosed wheeze [11]. Although confounding by indication has been repeatedly speculated to account for this observed association, emerging data suggests that this link is unlikely to be due to unmeasured confounding [12, 13]. For instance, prenatal and infant exposures to acetaminophen are associated with asthma development in childhood independent of factors that are indicative for acetaminophen use (e.g., pain, fever, and respiratory tract infections/influenza) [13]. The causal inference is further strengthened by results of meta-analyses [14–17] and by the observation of effect modification, in which maternal antioxidant gene polymorphisms modified the association between maternal use of acetaminophen during pregnancy and child risk of asthma [18]. The epidemiologically-observed association between acetaminophen use and asthma is biologically plausible and explained by the ability of acetaminophen to deplete glutathione, a key airway antioxidant [19]. Glutathione depletion has been shown to induce oxidative stress and promote inflammatory responses in the lungs [20, 21].

Sex differences in pharmacokinetics and pharmacodynamics of drugs, including acetaminophen, are widely recognized and their better understanding is necessary to optimize therapeutic doses [22]. Similarly, sex differences in the prevalence, incidence, and severity of a wide range of diseases, including asthma, have been documented. Specifically, a switchover in the prevalence of asthma, from male to female predominance, is observed during adolescence [23, 24]. In addition, murine studies have shown sex differences in susceptibility to acetaminophen-induced hepatotoxicity, with female mice demonstrating lower susceptibility than males [25–28]. Although the precise underlying mechanisms for the aforementioned sex differences are not understood, it has been demonstrated that female mice experience earlier recovery of hepatic and mitochondrial glutathione levels after glutathione depletion by acetaminophen [25, 26]. The early recovery of glutathione levels in female mice is speculated to contribute to their resistance to acetaminophen-induced hepatotoxicity. In the respiratory tract, previous animal model investigations demonstrated that acetaminophen, although at supratherapeutic doses, induced oxidative stress in the airways and caused injury in the nasal and lung tissues [27, 28], though sex differences were not addressed.

As males and females may have differential acetaminophen responses and subsequent differential asthma risk, this investigation sought to determine whether acetaminophen associates with asthma in a sex-specific manner. Such knowledge will add to the sex-stratified epidemiologic research by identifying those who will have the least or most harm from acetaminophen in regard to asthma risk. Furthermore, to better understand some of the varying biological factors underlying the sex-specific effect, we further hypothesized that leptin (*LEP*) and leptin receptor (*LEPR*) gene polymorphisms modulate the sex-specific association. This a priori conjecture is based on the observations that (i) leptin is associated with asthma [29], presumably through inducing oxidative stress and inflammation in the airways [30, 31], (ii) leptin exhibits a strong sex-related dimorphism, as females have higher concentrations compared to males [31, 32], and (iii) leptin mRNA expression is modulated in response to acetaminophen exposure [33, 34]. Leptin, a pleiotropic adipocyte-derived hormone with a key role in immune homeostasis and inflammatory responses, exerts its biological effects by binding to the leptin receptor, which is abundantly expressed in the lungs [31, 32]. Hence, using data from two population-based studies, we sought to determine whether sex modifies the association between acetaminophen use and asthma among young adults. Subsequently, we tested whether polymorphisms in the *LEP* and *LEPR* genes further modulate the identified sex-specific associations.

## Methods

### Kuwait University allergy (KUA) study

#### Study population

A cross-sectional study was conducted among students enrolled at Kuwait University (KU; *n* = 1154, from January to May 2015) [35]. The study was approved by the Health Sciences Center Ethical Committee at KU. Written informed consent was obtained from all study participants. Upon consenting, study participants were asked to self-complete a questionnaire that included questions on sociodemographic and lifestyle factors, and adapted the core items from the International Study of Asthma and Allergies in Childhood (ISAAC) questionnaire [36]. See Supplementary Methods in Additional file 1 for detailed information.

### Isle of Wight (IOW) birth cohort study

#### Study population

An unselected population-based birth cohort was recruited from all births (*n* = 1536) occurring between January 1989 and February 1990 on the Isle of Wight, UK, to prospectively study the natural history and etiology of allergic conditions [37]. Ethics approvals were obtained from the Local Research Ethics Committee and written informed consent was obtained from parents to enroll 1456 (95%) newborns, with follow-up assessments conducted at ages 1, 2, 4, 10, and 18 years. Participants and/or their parents completed study-specific and standardized (e.g., ISAAC) questionnaires [36, 37]. For the sake of comparison with the KUA study, the present report focuses on data collected at the 18 year follow-up (*n* = 1313). See Supplementary Methods in Additional file 1 for detailed information.

### Outcomes

In both the KUA and IOW studies, current asthma and current wheeze were ascertained based on self-report of symptoms and clinical history. Current asthma was defined by an affirmative response to the statements “history of physician-diagnosed asthma” and “wheezing in the past 12-months” and/or “asthma treatment in the past 12-months”. Current wheeze was defined by “wheezing in the past 12-months”.

### Exposures

In the KUA study, we asked a question adapted from the ISAAC questionnaire [36]: “In the past 12-months, how often, on average, have you taken acetaminophen (e.g., Panadol)?” Possible answer choices were: never (none in the past 12-months), medium (at least once in the past 12-months), and high (at least once per month in the past 12-months). In the IOW study, participants were asked to report average monthly use of acetaminophen, which then was categorized as never (none per month), medium (one or two times per month), and high (three or more times per month). Although the two studies have measured acetaminophen use differently, we used similar categories (never, medium, high frequency of use) to ease readability of the manuscript and presentation of tables.

### Covariates

In the IOW and KUA studies, information on covariates was obtained from questionnaires and physical assessments. Body mass index (BMI) was calculated as weight in kilograms divided by height in meters squared (kg/m^2^). BMI was modeled as a continuous variable. Current and past smoking status was self-reported by participants.

### *LEP* and *LEPR* genotyping

In the IOW study, single nucleotide polymorphisms (SNPs) spanning the *LEP* (k = 4) and *LEPR* (k = 14) genes were selected for genotyping based on a tagging scheme aiming to capture common, functional genetic variants that have been related to allergic conditions [38, 39]. Genomic DNA extracted from blood or saliva samples of 1211 study participants were interrogated using GoldenGate Genotyping Assays (Illumina, Inc., San Diego, CA) on the BeadXpress Veracode bead platform (Illumina, Inc., San Diego, CA) per Illumina’s protocol (see Supplementary Methods in Additional file 1). No genetic information was available in the KUA study.

### Statistical analysis

All statistical analyses were performed using SAS 9.4 (SAS Institute, Cary, North Carolina, USA). Adjusted prevalence ratios (PRs) and their 95% confidence intervals (CIs) were estimated using log-linear models with Poisson distribution and log-link accompanied by robust variance estimation, via the GENMOD procedure in SAS 9.4 [40]. Associations between acetaminophen use and current asthma and current wheeze were assessed. To determine whether sex is an effect modifier, interactions were evaluated on a multiplicative scale by including a product term (sex × acetaminophen use) in regression models. In the presence of statistical interaction, sex-stratified associations were assessed. All multivariable models were adjusted for sex, age, body-mass index (BMI), and current smoking status. The statistical significance level was set to α = 0.05 for all association and interaction assessments.

In the IOW study, the availability of genetic data enabled us to further assess the contribution of SNPs in *LEP* and *LEPR* genes to the effects of sex and acetaminophen use on current asthma. Deviation from Hardy-Weinberg Equilibrium was tested for each of the genotyped SNPs in *LEP* and *LEPR* genes using goodness-of-fit χ^2^ tests (see Additional file 1: Table S1) and estimates of linkage disequilibrium (LD) between SNPs were calculated using D′ and r^2^ measures (see Additional file 1: Figure S1 and Figure S2). Next, focusing on current asthma as an outcome, we screened for three-way multiplicative statistical interactions between *LEP* and *LEPR* SNPs (assuming linear increase in disease risk across genotypes, i.e., additive genetic risk model), sex, and acetaminophen use (assuming dose-effect: no, medium, and high use) on the risk of current asthma (product terms: *LEP* and *LEPR* SNP × sex × acetaminophen use). We evaluated 18 models to determine if there were three-way multiplicative statistical interactions (see Additional file 1: Table S2). To account for false findings due to multiple testing, we controlled the false discovery rate (FDR) [41]. In the presence of three-way multiplicative interactions, stratified analyses by the respective SNP genotypes were performed to describe the identified “sex × acetaminophen use” interaction across genotypes. To avoid small numbers within subgroups when assessing genotype-stratified associations, we applied a dominant genetic risk model to all SNPs, where the heterozygous and variant homozygous genotypes were combined together and assumed to be the risk group and the wild-type (most common) genotype was the reference group. The statistical significance for the FDR-adjusted *p*-values and for all association analyses was set to α = 0.05.

## Results

### Description of study populations

The KUA study enrolled 1154 students out of the 1561 students who were approached (response: 73.9%). In the IOW study, 1313 subjects participated in the 18-year follow-up, of whom 1305 had information on asthma symptoms and were analyzed in the current report. The median age of participants was 20.0 years in the KUA study and 17.8 years in the IOW study (Table 1). The prevalence of current asthma was 11.9% and 17.7% in the KUA and IOW studies, respectively. Current wheeze was reported by 14.6% and 22.2% of the KUA and IOW study participants, respectively. In regard to sex differences, in both studies females reported higher use of acetaminophen than males (Table 2). In the IOW study, the prevalence of current asthma and wheeze was higher in females compared to males. In contrast, current asthma and wheeze were higher among males compared to females in the KUA study (Table 2).

**Table 1:** Characteristics of participants of the Isle of Wight cohort study and Kuwait University Allergy study

	Isle of Wight study 18-year follow-up	Kuwait University Allergy study
Sex, % (n/total)		
Female	50.5 (659/1305)	77.3 (892/1154)
Male	49.5 (646/1305)	22.7 (262/1154)
Age (years)		
Median (5th, 95th percentile)	17.8 (17.2, 19.1)	20.0 (18.0, 26.0)
Body Mass Index (kg/m^2^)		
Median (5th, 95th percentile)	22.2 (18.2, 32.1)	23.4 (18.0, 33.3)
Current tobacco smoking, % (n/total)		
Yes	28.6 (364/1271)	14.2 (163/1151)
Acetaminophen use in the past 12-months, % (n/total)		
Never	NA	14.8 (170/1149)
Medium (at least once in the past year)	NA	31.9 (367/1149)
High (at least once per month)	NA	53.3 (612/1149)
Acetaminophen use per month, % (n/total)		
Never	45.3 (575/1270)	NA
Medium (one or two times per month)	37.9 (481/1270)	NA
High (three or more times per month)	16.8 (214/1270)	NA
Current wheeze, % (n/total)		
Yes	22.2 (290/1304)	14.6 (161/1101)
Current asthma, % (n/total)		
Yes	17.7 (231/1305)	11.9 (135/1135)

Figures are presented as % (n/total), except for age and body mass index, which were presented as median (5th, 95th percentile). NA: Not applicable (i.e., the question was not asked in the respective study)

**Table 2:** Prevalence of exposure and outcome variables in the Isle of Wight cohort study and Kuwait University Allergy study stratified by sex

	IOW Study, % (n/total)			KUA Study, % (n/total)		
	Males	Females	*P*	Males	Females	*P*
Acetaminophen use^a^						
Never	55.2 (348/630)	35.5 (227/640)	< 0.001	20.7 (54/261)	13.1 (116/888)	< 0.001
Medium	33.7 (212/630)	42.0 (269/640)		39.5 (103/261)	29.7 (264/888)	
High	11.1 (70/630)	22.5 (144/640)		39.8 (104/261)	57.2 (508/888)	
Current Wheeze						
Yes	18.9 (122/646)	25.5 (168/658)	0.004	19.2 (49/255)	13.2 (112/846)	0.018
Current Asthma						
Yes	15.9 (103/646)	19.4 (128/659)	0.099	15.2 (39/257)	10.9 (96/878)	0.065

*IOW* Isle of Wight study, *KUA* Kuwait University Allergy study

^a^In the IOW study, average times of acetaminophen use per month was reported and categorized as: never = none per month, medium = one or two times per month, and high = three or more times per month. In the KUA study, acetaminophen use in the past 12-months was reported as: never = none in the past 12-months, medium = at least once in the past12-months, and high = at least once per month in the past 12-months

### Association of acetaminophen use with asthma and wheeze and effect modification by sex

In IOW and KUA subjects, acetaminophen use was associated with a dose-dependent increased prevalence of current asthma (Table 3) and current wheeze (Table 4). Although the prevalence of wheeze and asthma differed among males and females and in KUA and IOW, the sex-stratified analysis showed that acetaminophen use at any level was associated with increased prevalence of current asthma and wheeze among male participants in both the IOW and KUA studies (Table 3 and Table 4). This sex-related effect modification was supported by a statistical interaction on a multiplicative scale. For example, in the IOW study, medium and high vs. no acetaminophen use was associated with current asthma among males (aPR: 1.88 and 3.99), but not (or to a lesser extent) among females (aPR: 0.94 and 1.59; interaction-term *p*-value = 0.012; Table 3).

**Table 3:** Associations between acetaminophen use and current asthma in the total population and stratified by sex: results from the Isle of Wight and Kuwait University Allergy studies

Acetaminophen use	Total Population		Males		Females		
	Current asthma, % (n/total)	Adjusted PR^c^ (95% CI)	Current asthma, % (n/total)	Adjusted PR^d^ (95% CI)	Current asthma, % (n/total)	Adjusted PR^d^ (95% CI)	*P*_interaction_^e^
IOW Study^a^							
Never	13.0 (75/575)	1.00	10.6 (37/348)	1.00	16.7 (38/227)	1.00	0.012
Medium	16.6 (80/481)	1.34 (0.96–1.88)	17.0 (36/212)	1.88 (1.17–3.01)	16.4 (44/269)	0.94 (0.60–1.46)	
High	32.2 (69/214)	2.43 (1.71–3.46)	40.0 (28/70)	3.99 (2.48–6.43)	28.5 (41/144)	1.59 (1.01–2.49)	
KUA Study^b^							
Never	8.4 (14/167)	1.00	5.7 (3/53)	1.00	9.7 (11/114)	1.00	0.032
Medium	11.4 (41/359)	1.35 (0.74–2.47)	19.0 (19/100)	3.25 (1.04–10.24)	8.5 (22/259)	0.85 (0.41–1.74)	
High	13.2 (80/606)	1.54 (1.02–2.79)	16.4 (17/104)	2.57 (1.01–8.28)	12.6 (63/502)	1.19 (0.64–2.23)	

*IOW* Isle of Wight study, *KUA* Kuwait University Allergy study, *PR* Prevalence ratio, *CI* Confidence interval

^a^Average times of acetaminophen use per month was reported and categorized as: never = none per month, medium = one or two times per month, and high = three or more times per month

^b^Acetaminophen use in the past 12-months was reported as: never = none in the past 12-months, medium = at least once in the past 12-months, and high = at least once per month in the past 12-months

^c^Adjusted for sex, age, body mass index, and current smoking status

^d^Adjusted for age, body mass index, and current smoking status

^e^Refers to the *p*-value associated with the interaction (product) term: ‘sex × acetaminophen use’

**Table 4:** Associations between acetaminophen use and current wheeze in the total population and stratified by sex: results from the Isle of Wight and Kuwait University Allergy studies

Acetaminophen use	Total Population		Males		Females		
	Current wheeze, % (n/total)	Adjusted PR^c^ (95% CI)	Current wheeze, % (n/total)	Adjusted PR^d^ (95% CI)	Current wheeze, % (n/total)	Adjusted PR^d^ (95% CI)	*P*_interaction_^e^
IOW Study^a^							
Never	15.8 (91/575)	1.00	13.2 (46/349)	1.00	19.9 (45/226)	1.00	0.011
Medium	23.0 (106/482)	1.36 (1.02–1.81)	21.1 (45/213)	1.80 (1.19–2.71)	22.7 (61/269)	1.01 (0.67–1.47)	
High	38.9 (84/216)	2.34 (1.73–3.16)	43.7 (31/71)	3.60 (2.36–5.49)	36.6 (53/145)	1.63 (1.12–2.39)	
KUA Study^b^							
Never	8.6 (14/163)	1.00	5.8 (3/52)	1.00	9.9 (11/111)	1.00	0.064
Medium	14.8 (51/345)	1.82 (0.99–3.33)	21.8 (22/101)	3.27 (1.04–10.31)	11.9 (29/244)	1.26 (0.61–2.61)	
High	16.2 (96/591)	2.12 (1.19–3.80)	23.5 (24/102)	3.63 (1.16–11.31)	14.7 (72/489)	1.66 (0.86–3.22)	

*IOW* Isle of Wight study, *KUA* Kuwait University Allergy study, *PR* Prevalence ratio, *CI* Confidence interval

^a^Average times of acetaminophen use per month was reported and categorized as: never = none per month, medium = one or two times per month, and high = three or more times per month

^b^Acetaminophen use in the past 12-months was reported as: never = none in the past 12-months, medium = at least once in the past 12-months, and high = at least once per month in the past 12-months

^c^Adjusted for sex, age, body mass index, and current smoking status

^d^Adjusted for age, body mass index, and current smoking status

^e^Refers to the p-value associated with the interaction (product) term: ‘sex × acetaminophen use’

### *LEP*/*LEPR* SNPs, sex, and acetaminophen use in relation to asthma

**Fig. 1:**
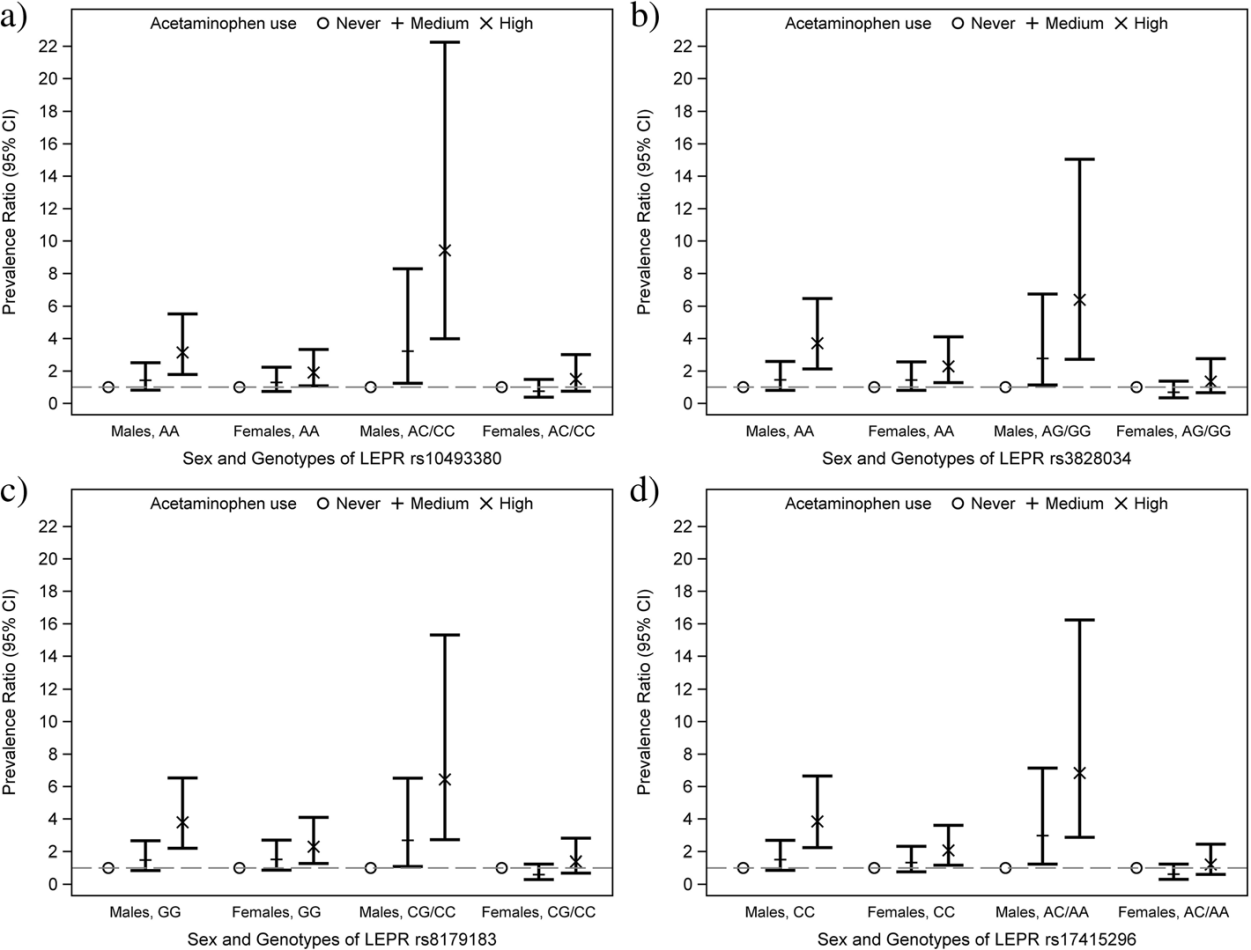
Associations between acetaminophen use and current asthma stratified by sex and genotypes of leptin receptor (*LEPR*) gene polymorphisms: results from the Isle of Wight study. A) Associations stratified by sex and genotypes of *LEPR* rs10493380. B) Associations stratified by sex and genotypes of *LEPR* rs3828034. C) Associations stratified by sex and genotypes of *LEPR* rs8179183. D) Associations stratified by sex and genotypes of *LEPR* rs17415296

In analysis of the IOW cohort, we explored whether the identified sex-related effect modification of the acetaminophen-asthma association varied across *LEP* and *LEPR* genotypes. First, we screened for three-way interactions on a multiplicative scale. After adjusting for multiple testing, the screening process revealed the presence of three-way interactions between four *LEPR* SNPs, sex, and acetaminophen use on the risk of current asthma (see Additional file 1: Table S2). Stratified analysis by genotypes of *LEPR* SNPs showed that the sex-related modifications were present only among participants who carried one or two copies of the *LEPR* SNP minor alleles (rs10493380, rs3828034, rs8179183, rs17415296; see Additional file 1: Table S3). For instance, there was a “sex × acetaminophen use” interaction on a multiplicative scale among participants carrying the AC or CC genotypes of rs10493380 (interaction-term *p*-value < 0.001); however, there was no evidence for a “sex × acetaminophen use” interaction on a multiplicative scale among subjects with the AA genotype (interaction-term p-value = 0.428; see Additional file 1: Table S3). Moreover, sex- and genotype-stratified analysis indicated that acetaminophen use was associated with asthma to a similar extent among male and female participants carrying two wild-type alleles of *LEPR* SNPs. In contrast, among those carrying at least one copy of the minor allele for *LEPR* SNPs, the magnitude of the acetaminophen use effect on asthma was highly pronounced among males (rs17415296: aPR = 6.83,), but not among females (rs17415296: aPR = 1.22; Fig. 1, see Additional file 1: Table S3).

## Discussion

This investigation, using data from two population-based observational studies, for the first time showed that sex modified the association between acetaminophen use and current asthma and wheeze among young adults. Sex-stratified analysis indicated that acetaminophen use was associated with increased prevalence of current asthma and wheeze among males, but not (or to a lesser extent) in female participants. This heterogeneity in the effect across sexes was supported by the presence of statistical interaction on a multiplicative scale. Furthermore, we demonstrated for the first time that genotypes of *LEPR* SNPs modified the observed sex-specific acetaminophen-asthma association. In particular, the sex-related effect modification was confined to participants who carried one or two copies of the *LEPR* SNPs minor alleles; whereas, acetaminophen use was associated with asthma to a similar magnitude of effect among males and females who were homozygous for the wild-type allele of *LEPR* SNPs. Hence, the observed sex-specific association between acetaminophen use and asthma appeared to be limited to individuals carrying certain *LEPR* genotypes.

Our interest in whether sex plays a role in the acetaminophen-asthma association is based on the fact that sex has a central role in the natural history of asthma [23] and the observation of sex-dependent acetaminophen-induced hepatotoxicity in murine models, with female mice demonstrating lower susceptibility than male mice [25, 26]. In support of our hypothesis, we have demonstrated that acetaminophen use was associated with increased prevalence of current asthma and wheeze among male participants in both the IOW and KUA studies, but not (or to a lesser extent) in female participants. A report based on the ISAAC study conducted among children aged 6–7 years showed that acetaminophen use was associated with current asthma symptoms to a similar magnitude of effect among male (OR = 1.74, 95% CI: 1.63–1.85) and female (OR = 1.79, 95% CI: 1.67–1.92) subjects [7]. Surprisingly, the ISAAC report on the acetaminophen-asthma association among the 13–14 year old children did not address sex differences [8].

Prior investigations reported effect modification of the acetaminophen-asthma association by genetic variants [18, 42–44]. We demonstrated that acetaminophen use increased the risk of asthma to a similar magnitude in males and females who carry two copies of the wild-type allele of *LEPR* SNPs. Whereas, carrying at least one copy of the minor allele of *LEPR* SNPs lead to a sex-related differential effect of acetaminophen use on asthma risk (Fig. 1). Our a priori interest in the leptin and leptin receptor pathway is multifold. First, leptin and leptin receptor are expressed in lung tissue and their expression levels have been shown to be associated with asthma development and severity [29, 45]. Second, leptin expression exhibits strong sex-related differences that are not entirely explained by sex hormones nor sex-dependent distribution of body fat [31, 32]. Third, acetaminophen exposure modulates leptin expression as measured in peripheral blood [33, 34]. Fourth, negative correlation between leptin and glutathione levels have been reported [46–48]. An in vitro study showed that stimulating experimental cells with leptin induced oxidative stress and pro-inflammatory responses [30]. Of note, the importance of the leptin receptor is demonstrated through its (i) association with leptin, (ii) expression in the lung, and (iii) role in immune functions [31, 32, 49]. Hence, leptin may have the potential to augment and/or suppress the pulmonary response to acetaminophen. Our results suggest that leptin, acting thought its receptor, mediates the association between acetaminophen use and asthma through its involvement in inducing oxidative stress in the lung. Moreover, the proposed mechanism of action linking acetaminophen use and leptin/leptin receptor on asthma risk seems to be further modulated by sex. Our observational findings of sex- and *LEPR*-dependent associations need further exploration to understand the biological factors underlying the sex- and genotype-specific effects.

A major strength of our study is observing a consistent sex-specific association in two temporally-spaced studies that enrolled geographically and culturally different groups of individuals; this indicates the robustness of our findings. Furthermore, the large sample sizes of the IOW and KUA studies provided sufficient statistical power to detect interaction terms (higher-order terms). The longitudinal design of the IOW birth cohort study that prospectively ascertained asthma status is an added strength.

The possibility of a selection bias due to attrition is unlikely due to the high follow-up proportion at age 18-years (90.2%) in the IOW study [37]. Similarly, self-selection bias was not a great concern in the KUA study since the response proportion was high (73.9%) and the sex distribution in the study sample (77.3% females and 22.7% males) closely resembled the sex distribution in the target population of the KU student body (73.5% females and 26.5% males) [35]. The KUA study did not include genotyping so replication of genetic findings was not pursued.

Current asthma and wheeze were defined using the same criteria in both the IOW and KUA studies, which minimized inter-studies phenotype heterogeneity. Although levels of acetaminophen use were differentially ascertained in the two studies, we observed a consistent sex-related effect modification. Sensitivity analysis aiming to reduce inter-studies heterogeneity in ascertaining exposure by dichotomizing acetaminophen use to ‘never’ and ‘any use’ showed similar results to our original findings (see Additional file 1: Table S4). Confounding by indication did not completely account for the observed acetaminophen-asthma association [12, 13]; however, the effect of reverse causality cannot be ruled-out in the current report since acetaminophen use and asthma were ascertained concurrently in the two analyzed studies. The observed sex and *LEPR* gene effect modification, though, is unlikely to be explained by confounding by indication and is suggestive of a potential causal association.

## Conclusions

The findings of this study add to exiting knowledge by demonstrating that the acetaminophen-asthma association could be sex- and genotype-specific. Acetaminophen appears to be associated with asthma risk in a sex-specific manner with young adult males, but not females, being more susceptible. We also demonstrated that the sex-specific association was confined to participants who carry one or two copies of the *LEPR* SNPs minor alleles, where the acetaminophen use effect was pronounced among males but not among females. In contrast, among individuals homozygous for the common allele of *LEPR* SNPs, acetaminophen associated with asthma among males and females to a similar magnitude of effect, indicating that acetaminophen effect was modulated by sex and genetic make-up. If the acetaminophen-asthma association is causal, then our findings will aid susceptibility-based stratification of at-risk individuals and augment preventive public health efforts. Future studies are needed to corroborate and extend our findings.

## Additional file

Additional file 1: Supplementary Material. Kuwait University Allergy (KUA) study – Study setting and population. Isle of Wight (IOW) birth cohort study – Study setting and population. *LEP* and *LEPR* genotyping – IOW study. Additional file: Table S1**.** Genotype and minor allele frequencies of leptin (*LEP*) and leptin receptor (*LEPR*) gene single nucleotide polymorphisms: results from the Isle of Wight study. Additional file**:**Table S2. Evaluating three-way statistical interactions on a multiplicative scale between sex, acetaminophen use and polymorphisms in leptin (*LEP*) and leptin receptor (*LEPR*) genes on the risk of current asthma: results from the Isle of Wight study. Additional file 1: Table S3. Associations between acetaminophen use and current asthma stratified by sex and genotypes of leptin receptor (*LEPR*) gene polymorphisms: results from the Isle of Wight study. Addtional file 1: Table S4. Associations between acetaminophen use and current asthma in the total population and stratified by sex: results of sensitivity analysis from the Isle of Wight and Kuwait University Allergy studies. Additional file 1: Figure S1. Estimates of linkage disequilibrium (LD) between leptin (*LEP*) gene single nucleotide polymorphisms. (A) LD estimates using *D*′ values and (B) LD estimates using *r*^2^ values: results from the Isle of Wight study. Additional file 1: Figure S2. Estimates of linkage disequilibrium (LD) between leptin receptor (*LEPR*) gene single nucleotide polymorphisms. (A) LD estimates using *D*′ values and (B) LD estimates using *r*^2^ values: results from the Isle of Wight study. (PDF 147 kb)

## Abbreviations

BMI: Body mass index
CI: Confidence intervals
FDR: False discovery rate
IOW: Isle of Wight study
ISAAC: International Study of Asthma and Allergies in Childhood
KUA: Kuwait University Allergy study
LD: Linkage disequilibrium
LEP: Leptin gene
LEPR: Leptin receptor gene
mRNA: Messenger Ribonucleic acid
PR: Prevalence ratio
SNPs: Single nucleotide polymorphisms
