# Supplementary material for: Leptin receptor gene polymorphisms and sex modify the association between acetaminophen use and asthma among young adults: results from two observational studies
Source: Respir Res. 2018 Sep 19;19:179. doi: 10.1186/s12931-018-0892-y (PMC6146615; doi:10.1186/s12931-018-0892-y)
Supplement: Supplementary file 1 — Supplementary Material. Kuwait University Allergy (KUA) study – Study setting and population. Isle of Wight (IOW) birth cohort study – Study setting and population. LEP and LEPR genotyping – IOW study. Additional file: Table S1. Genotype and minor allele frequencies of leptin (LEP) and leptin receptor (LEPR) gene single nucleotide polymorphisms: results from the Isle of Wight study. Additional file:Table S2. Evaluating three-way statistical interactions on a multiplicative scale between sex, acetaminophen use and polymorphisms in leptin (LEP) and leptin receptor (LEPR) genes on the risk of current asthma: results from the Isle of Wight study. Additional file 1: Table S3. Associations between acetaminophen use and current asthma stratified by sex and genotypes of leptin receptor (LEPR) gene polymorphisms: results from the Isle of Wight study. Addtional file 1: Table S4. Associations between acetaminophen use and current asthma in the total population and stratified by sex: results of sensitivity analysis from the Isle of Wight and Kuwait University Allergy studies. Additional file 1: Figure S1. Estimates of linkage disequilibrium (LD) between leptin (LEP) gene single nucleotide polymorphisms. (A) LD estimates using D′ values and (B) LD estimates using r2 values: results from the Isle of Wight study. Additional file 1: Figure S2. Estimates of linkage disequilibrium (LD) between leptin receptor (LEPR) gene single nucleotide polymorphisms. (A) LD estimates using D′ values and (B) LD estimates using r2 values: results from the Isle of Wight study. (PDF 147 kb) [file 12931_2018_892_MOESM1_ESM.pdf]

Supplementary Material

Manuscript title: Leptin receptor gene polymorphisms and sex modify the association between acetaminophen use and asthma among young adults: results from two observational studies

Authors: Ali H. Ziyab,* Nandini Mukherjee, Ramesh J. Kurukulaaratchy, Hongmei Zhang, Susan Ewart, Hasan Arshad, Wilfried Karmaus

*** Corresponding author:**

Ali H. Ziyab, PhD

Departments of Community Medicine and Behavioral Sciences, Faculty of Medicine, Kuwait University, Kuwait.

Tel: (+965) 24636545

Fax: (+965) 25338948

Email: aziyab@hsc.edu.kw

Supplementary Methods

Kuwait University Allergy (KUA) study – Study setting and population

A cross-sectional study was conducted among students enrolled at Kuwait University (KU), which represents the only public university and the largest higher-education institution in Kuwait; enrolling approximately 36 000 undergraduate and graduate students with a female-to-male ratio of around 3:1. The current study enrolled 1154 students during the period from January to May, 2015. KU consists of 15 colleges scattered across five campuses. This study recruited students from the five campuses of KU using proportional allocation to the size strategy through estimating the number of students (participants) needed from each campus relative to the entire population of students at KU. Hence, campuses with larger student body received more weight in the study sample size compared to smaller campuses; increasing the representativeness of the study sample. A random sample of instructors from each of the five campuses were emailed and asked about their willingness to allow the research team to introduce the study and distribute the questionnaire to students. Around 65% replied with positive responses. Students who were available at the selected classrooms were invited to participate; thus, study participants were recruited using a convenience sampling. Students aged ≥ 18 years old who were registered at the different colleges of KU at the time of the study were eligible to participate in the study. The study was approved by the Health Sciences Center Ethical Committee at KU. Written informed consent was obtained from all study participants. Upon consenting, study participants were asked to self-complete a questionnaire that included questions on sociodemographic and lifestyle factors, maternal and paternal history of allergic diseases, and adapted the core items from the ISAAC questionnaire regarding symptoms of asthma, rhinitis, and eczema

Isle of Wight (IOW) birth cohort study – Study setting and population

An unselected population-based birth cohort was recruited from all births (n = 1536) occurring between January 1989 and February 1990 on the Isle of Wight, UK, to prospectively study the natural history and etiology of allergic conditions. After exclusion of adoptions, perinatal deaths, and refusal for follow-up, written informed consent was obtained from parents to enroll 1456 (95%) newborns, with follow-up assessments conducted at 1, 2, 4, 10, and 18 years of age. Ethics approvals were obtained from the Isle of Wight Local Research Ethics Committee (recruitment, 1, 2 and 4 years) and National Research Ethics Service, NRES Committee South Central – Southampton B (10 and 18 years) (06/Q1701/34). Participants and/or their parents completed study-specific and standardized (e.g., International Study of Asthma and Allergies in Childhood [ISAAC]) questionnaires. The present report focuses on data collected at the 18 year follow-up (n=1313).

***LEP* and *LEPR* genotyping – IOW study**

The genotype tagging system scheme used to tag the SNPs gave priority to the variants that showed strong association with asthma in the Isle of Wight birth cohort, or had been reported by others to be associated with asthma, or allergy and have functional importance (non-synonymous, located in conserved regions of DNA or present in regulatory regions). *LEP* and *LEPR* SNPs (including 10 kb upstream and downstream of the gene) were determined using the tagger implemented in Haploview v3.2 based on Caucasian HapMap data. The threshold value for r^2 was taken as 0.2 for tagging and one, two, and three SNP marker combination tests were used. Four *LEP* SNPs (rs4731429, rs10249476, rs10954176, and rs11763517) and fourteen *LEPR* SNPs (rs1137100, rs1137101, rs1171275, rs1782754, rs3762274, rs3790424, rs3806318,

rs3828034, rs6678033, rs7526141, rs8179183, rs10493380, rs12059300, and rs17415296) were selected. DNA extracted from blood or saliva samples of 1211 cohort subjects were interrogated using GoldenGate Genotyping Assays (Illumina Inc., San Diego, CA) on the Bead Xpress Veracode platform per Illumina's protocol. DNA from each subject plus 37 replicate samples were analyzed. The quality threshold for allele determination was set at a GenCall score >0.25 with 98.3% retained for further analysis. Analysis of each locus included reclustering of genotyping data using our project data to define genotype cluster positions with additional manual reclustering to maximize both cluster separation and the 50th percentile of the distribution of the GenCall scores across all genotypes (50% GC score).

Table S1. Genotype and minor allele frequencies of leptin (*LEP*) and leptin receptor (*LEPR*) gene single nucleotide polymorphisms: results from the Isle of Wight study

SNP	Genotypes	Genotype frequencies (n)	Minor allele frequency	HWE *P* value
LEP				
rs4731429	GG/AG/AA	332/582/238	0.46	0.586
rs10249476	CC/AC/AA	453/542/156	0.37	0.822
rs10954176	AA/AG/GG	338/534/271	0.47	0.039
rs11763517	AA/AG/GG	293/569/289	0.49	0.757
LEPR				
rs1137100	AA/AG/GG	592/465/97	0.29	0.713
rs1137101	AA/AG/GG	347/558/246	0.46	0.486
rs1171275	GG/AG/AA	784/334/32	0.17	0.696
rs1782754	AA/AG/GG	590/473/98	0.29	0.834
rs3762274	AA/AG/GG	421/525/167	0.39	0.963
rs3790424	AA/AG/GG	580/457/107	0.29	0.242
rs3806318	AA/AG/GG	580/465/95	0.29	0.909
rs3828034	AA/AG/GG	777/330/39	0.18	0.640
rs6678033	GG/AG/AA	447/533/159	0.37	0.999
rs7526141	GG/AG/AA	341/556/256	0.46	0.338
rs8179183	GG/CG/CC	787/320/37	0.17	0.576
rs10493380	AA/AC/CC	791/317/31	0.17	0.999
rs12059300	GG/AG/AA	741/378/37	0.20	0.206
rs17415296	CC/AC/AA	793/332/38	0.18	0.717

HWE: Hardy-Weinberg equilibrium; SNP: single nucleotide polymorphism

Table S2. Evaluating three-way statistical interactions on multiplicative scale between sex, acetaminophen use and polymorphisms in leptin (*LEP*) and leptin receptor (*LEPR*) genes on the risk of current asthma: results from the Isle of Wight study

**Interaction (product) term***	$P_{\text{interaction}}$	**FDR adjusted** $P_{\text{interaction}}$
LEP		
Sex × acetaminophen use × rs4731429	0.868	0.992
Sex × acetaminophen use × rs10249476	0.801	0.992
Sex × acetaminophen use × rs10954176	0.889	0.992
Sex × acetaminophen use × rs11763517	0.292	0.584
LEPR		
Sex × acetaminophen use × rs1137100	0.414	0.745
Sex × acetaminophen use × rs1137101	0.735	0.992
Sex × acetaminophen use × rs1171275	0.937	0.992
Sex × acetaminophen use × rs1782754	0.465	0.761
Sex × acetaminophen use × rs3762274	0.096	0.291
Sex × acetaminophen use × rs3790424	0.518	0.777
Sex × acetaminophen use × rs3806318	0.113	0.291
Sex × acetaminophen use × rs3828034	0.004	**0.036**
Sex × acetaminophen use × rs6678033	0.177	0.398
Sex × acetaminophen use × rs7526141	0.997	0.997
Sex × acetaminophen use × rs8179183	0.008	**0.036**
Sex × acetaminophen use × rs10493380	0.0002	**0.004**
Sex × acetaminophen use × rs12059300	0.103	0.291
Sex × acetaminophen use × rs17415296	0.006	**0.036**

FDR: false discovery rate.

Figures in bold refer to interaction terms that remained statistically significant at α = 0.05 after controlling the false discovery rate.

*Acetaminophen use was modeled as a continuous variable with three increasing levels of use: never (coded: 0), medium (coded: 1), and high (coded: 2). Additive genetic risk model, i.e. assuming linear increase in disease risk across genotypes, was applied for all single nucleotide polymorphisms (SNPs). The three genotypes were coded and entered in the regression model in a dosage-effect manner (from smaller to larger effect). And sex was modeled as a binary variable (male/female).

Table S3. Associations between acetaminophen use and current asthma stratified by sex and genotypes of leptin receptor (*LEPR*) gene polymorphisms: results from the Isle of Wight study

SNP Genotype	acetaminophen use*	Males		Females		
		Current asthma, % (n/total)	Adjusted PR† (95% CI)	Current asthma, % (n/total)	Adjusted PR† (95% CI)	$P_{interaction}$⁋
**rs10493380**						
AA	Never	13.0 (24/185)	1.00	14.3 (19/133)	1.00	0.428
	Medium	16.7 (22/132)	1.43 (0.82-2.51)	17.6 (26/148)	1.29 (0.74-2.24)	
	High	35.6 (16/45)	3.14 (1.78-5.52)	26.7 (24/90)	1.90 (1.09-3.33)	
AC/CC	Never	6.8 (6/88)	1.00	23.4 (15/64)	1.00	< 0.001
	Medium	21.7 (10/46)	3.22 (1.25-8.29)	16.7 (13/78)	0.75 (0.38-1.48)	
	High	64.7 (11/17)	9.42 (3.99-22.25)	33.3 (10/30)	1.51 (0.75-3.02)	
**rs3828034**						
AA	Never	12.6 (23/182)	1.00	12.5 (17/136)	1.00	0.465
	Medium	16.3 (20/123)	1.45 (0.81-2.59)	17.2 (26/151)	1.44 (0.81-2.56)	
	High	40.5 (17/42)	3.71 (2.13-6.47)	27.9 (24/86)	2.29 (1.28-4.11)	
AG/GG	Never	7.4 (7/95)	1.00	24.1 (14/58)	1.00	0.006
	Medium	20.4 (11/54)	2.77 (1.14-6.75)	15.9 (13/82)	0.69 (0.35-1.38)	
	High	47.4 (9/19)	6.39 (2.72-15.05)	31.3 (10/32)	1.35 (0.66-2.76)	
**rs8179183**						
GG	Never	12.6 (23/183)	1.00	12.4 (17/137)	1.00	0.400
	Medium	16.7 (21/126)	1.50 (0.85-2.66)	18.2 (28/154)	1.53 (0.87-2.70)	
	High	40.9 (18/44)	3.79 (2.20-6.53)	27.6 (24/87)	2.29 (1.28-4.10)	
CG/CC	Never	7.8 (7/90)	1.00	24.1 (14/58)	1.00	0.008
	Medium	20.8 (11/53)	2.68 (1.10-6.52)	13.8 (11/80)	0.60 (0.29-1.24)	
	High	50.0 (9/18)	6.44 (2.71-15.31)	32.3 (10/31)	1.39 (0.69-2.82)	
**rs17415296**						
CC	Never	12.4 (23/186)	1.00	13.6 (19/140)	1.00	0.267
	Medium	16.5 (21/127)	1.52 (0.86-2.68)	17.5 (27/154)	1.34 (0.77-2.32)	
	High	40.9 (18/44)	3.85 (2.23-6.65)	27.3 (24/88)	2.06 (1.18-3.61)	
AC/AA	Never	7.4 (7/95)	1.00	25.4 (15/59)	1.00	0.002
	Medium	21.8 (12/55)	2.97 (1.24-7.13)	15.0 (12/80)	0.62 (0.31-1.24)	
	High	50.0 (9/18)	6.83 (2.87-16.24)	30.3 (10/33)	1.22 (0.61-2.45)	

SNP: single nucleotide polymorphism.

*Average times of acetaminophen use per month was reported and categorized as: never = none per month, medium = one or two times per month, and high = three or more times per month.

†Adjusted for age, body mass index, and current smoking status.

⁋Refers to the p-value associated with the interaction (product) term: 'sex × acetaminophen use'.

Table S4. Associations between acetaminophen use and current asthma in the total population and stratified by sex: results of sensitivity analysis from the Isle of Wight and Kuwait University Allergy studies

Acetaminophen use	Total Population		Males		Females		
	**Current asthma, % (n/total)**	**Adjusted PR† (95% CI)**	**Current asthma, % (n/total)**	**Adjusted PR‡ (95% CI)**	**Current asthma, % (n/total)**	**Adjusted PR‡ (95% CI)**	$P_{interaction}$⁋
**IOW Study***							
Never	13.0 (75/575)	1.00	10.6 (37/348)	1.00	16.7 (38/227)	1.00	0.015
Any use	21.4 (149/695)	1.65 (1.22-2.24)	22.7 (64/282)	2.39 (1.57-3.65)	20.6 (85/413)	1.15 (0.78-1.71)	
**KUA Study#**							
Never	8.4 (14/167)	1.00	5.7 (3/53)	1.00	9.7 (11/114)	1.00	0.048
Any use	12.5 (121/965)	1.47 (0.98-2.37)	17.7 (36/204)	2.85 (1.02-9.31)	11.2 (85/761)	1.08 (0.58-1.99)	

IOW: Isle of Wight study; KUA: Kuwait University Allergy study; PR: Prevalence ratio; CI: Confidence interval.

*Average times of acetaminophen use per month was reported and categorized as: never = none per month and any use = one or more times per month.

#Acetaminophen use in the past 12-months was reported as: never = none in the past 12-months and any use = once or more in the past 12-months.

†Adjusted for sex, age, body mass index, and current smoking status.

‡Adjusted for age, body mass index, and current smoking status.

⁋Refers to the p-value associated with the interaction (product) term: ‘sex × acetaminophen use’.

(A) Linkage disequilibrium using *D′* values

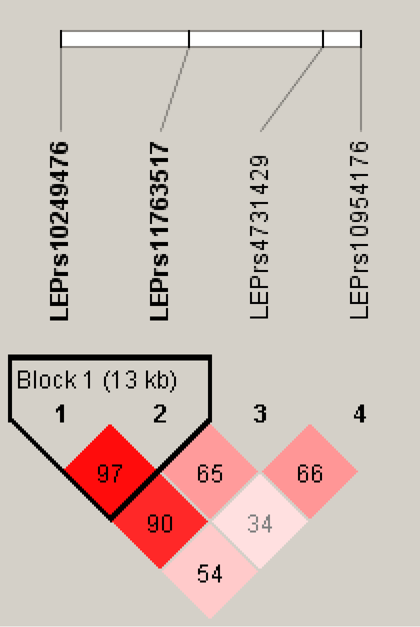

(B) Linkage disequilibrium using r^2 values

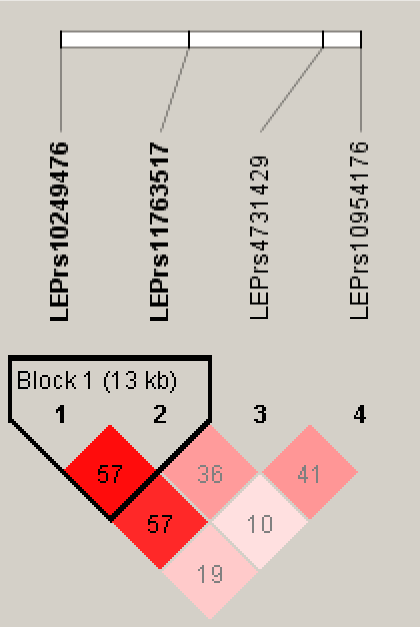

Fig. S1. Estimates of linkage disequilibrium (LD) between leptin (*LEP*) gene single nucleotide polymorphisms. (A) LD estimates using *D′* values and (B) LD estimates using r^2 values: results from the Isle of Wight study.

(A) Linkage disequilibrium using *D′* values

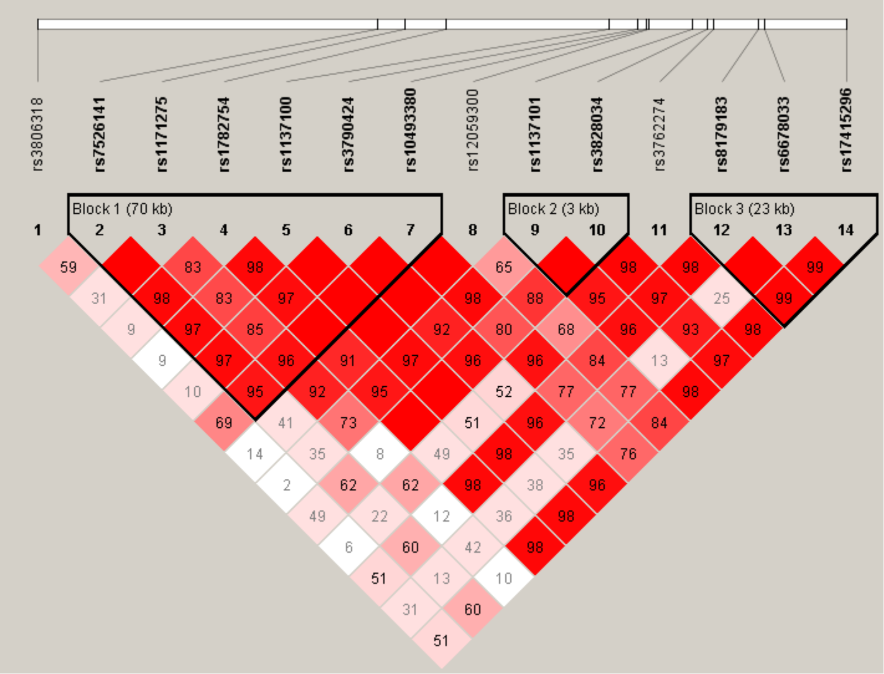

(B) Linkage disequilibrium using r^2 values

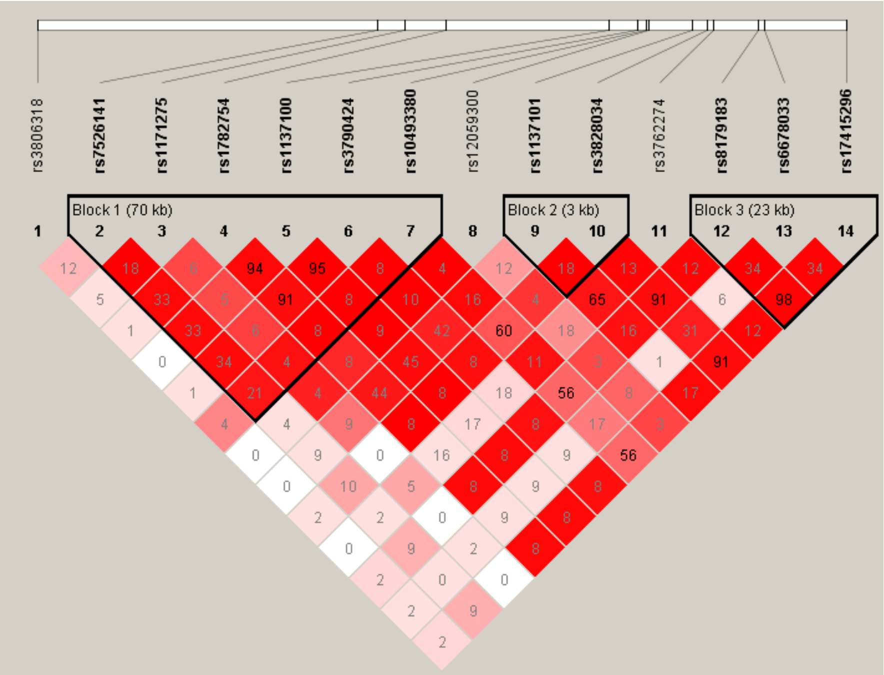

Fig. S2. Estimates of linkage disequilibrium (LD) between leptin receptor (*LEPR*) gene single nucleotide polymorphisms. (A) LD estimates using *D′* values and (B) LD estimates using r^2 values: results from the Isle of Wight study.
